# Potential human health risks associated with ingestion of heavy metals through fish consumption in the Gulf of Guinea

**DOI:** 10.1016/j.toxrep.2023.01.005

**Published:** 2023-01-16

**Authors:** Elvis Nyarko, Charles Mario Boateng, Obed Asamoah, Maurice Oti Edusei, Edem Mahu

**Affiliations:** a Department of Marine and Fisheries Sciences, University of Ghana, Legon, Ghana; b Regional Maritime University, Accra, Ghana

**Keywords:** Heavy metals, Fish, Carcinogenic risks, Humans, Ghana

## Abstract

Heavy metal pollution of the marine environment has toxic implications for both the aquatic biota and human health. We examined the levels of Zinc (Zn), Lead (Pb), Copper (Cu), Cadmium (Cd), Arsenic (As) and Mercury (Hg) in muscles of *Sardinella maderensis, Dentex angolensis, Sphyraena sphyraena and Penaeus notialis* caught from the coastal waters of Ghana using inductively coupled plasma mass spectrometry method. *Penaeus notialis* recorded the highest concentrations of all the metals (Cu:12.08 ± 1.46 µg/g, Zn: 19.20 ± 2.27 µg/g, As: 8.46 ± 2.42 µg/g, and Cd: 0.03 ± 0.01 µg/g) except Hg. Mercury was relatively high in *D. angolensis* (0.14 ± 0.03 µg/g). Apart from As, all metals were within globally permissible daily limits for consumption by human per meal. The estimated Target Hazard Quotient due to the intake of Hg through D. *angolensis* consumption exceeded the threshold value across all age categories. Carcinogenic risks due to As intake through *P. notialis* consumption far exceeded the 10^−6^ threshold for all age groups in Ghana*.* It is recommended that the consumption of these fish species particularly, the shrimp *P. notialis* be done cautiously to avoid possible future health challenges.

## Graphical Abstract

**Figure:**
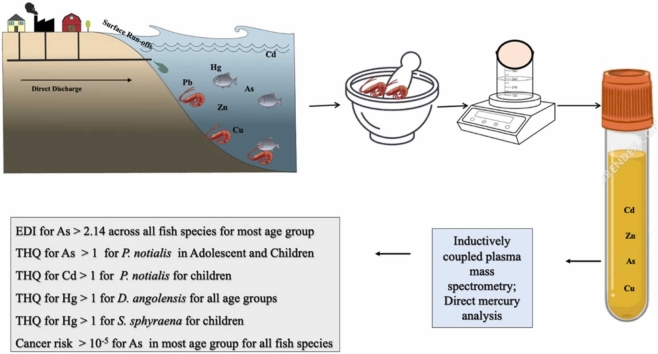

## 1 Introduction

Fish remains among the most traded food commodities and plays an important role in global food security and nutrition [20]. Since 1960, a remarkable surge has been observed in the world’s per capita apparent fish consumption, from an average of 9.9 kg in 2000 to around 20.4 kg in 2019 [23]. Ranked among the top fish-consuming countries in the world, Ghana’s nationwide fish consumption has been reported to hit one million metric tonnes annually. Fish is the cheapest source of protein, constituting more than 60% of the animal protein diet in several African countries [20].

Despite the far-reaching health benefits associated with fish consumption, the potential for it to bioaccumulate contaminants from the surroundings and pass on to humans makes its consumption a health threat in certain instances [18], [41], [42]. In Africa, the continuous discharge of contaminants such as heavy metals, PAHs, PCBs, plastics, pharmaceuticals and other waste substances into the marine environment has become a huge challenge with unclear management solutions. Human exposure to heavy metals should be of utmost concern to all because of the stable, non-biodegradable, persistent nature of these contaminants and their ability to accumulate in organs and tissues when exposed to them over a long period of time [1], [18], [42], [49], [50], [9]. Reported health risks emanating from metal toxicity to humans include but are not limited to kidney and skeletal damage, neurological disorders, endocrine disruption, cardiovascular dysfunction, and various cancers [25], [47]. Enrichment of Cu in the body may affect gastrointestinal, cardiovascular, hematological, hepatic, renal, and central nervous system functioning [47]. Likewise, elevated levels of Zn may induce symptoms such as vomiting, chest tightness, nausea, excitement, coldness, unconsciousness, coma or even death resulting from pulmonary edema and liver damage [47]. Prolonged exposure to Hg, Cd, and Pb has been reported to result in dermatitis, lung fibrosis, cardiovascular and kidney diseases, as well as lung and nasal cancers [4], [47]. To understand and minimize the potential health risks that may be associated with heavy metals through the consumption of seafood, global guidelines have been established to guide the safe consumption of fish [19]. Despite mounting evidence of increasing heavy metal pollution in coastal waters of developing countries, data on levels of heavy metals in fish, daily intake levels and possible health risks to humans are limited [2], [38], [39].

Globally, studies have been conducted to assess the potential health risks associated with heavy metals in foods consumed by humans, with some revealing negative health implications while others revealing no adverse health effects. For example, to establish the safety of baby foods and milk powders in Iran, Kiani et al. [32] evaluated the concentration and health risk of Cu, Fe, Zn, Ni, Hg, As, Cd, and Al in these food items. Their study concluded that the content of the toxic element in the tested products was sufficiently low and that all the milk powder and baby food sold in Iran could be considered safe for infants and children. Similarly, Karami et al. [30] reported no concern regarding the non-carcinogenic risk due to the ingestion of heavy metals via the consumption of edible mushrooms, except for Hg in wild mushrooms for children. Conversely, Fu et al. [26] found high cancer for As (1.01 × 10^−4^) in wild mushrooms from Yunnan Province in China. Meanwhile, Kumar et al. [34] reported adverse health risks for Hg and Cd in children through the consumption of finfish from the coastal waters of India. Ghana is not an exception to studies that have evaluated human health risks from the consumption of diverse food items, including vegetables, tuber crops, poultry, and fish. Bortey-Sam et al., ($year$) [11], [27], [33], [35], [37], [52], [6], [7]. Some of these studies concluded that heavy metals had implications for human health, while others found no adverse effects at all. Most of these studies have used the flame atomic adsorption spectrometry technique, which often presents detection limit challenges. It is necessary that other techniques are used to broaden and improve our understanding of the potential health risks of heavy metals in Ghana’s aquatic systems. The current study uses inductively coupled plasma mass spectrometric and direct mercury analysis techniques to determine the concentration of heavy metals in four commonly consumed marine fish species landed in Ghana. Estimate daily intake levels per heavy metal and evaluate the potential carcinogenic and non-carcinogenic risks associated with the consumption of these fish species across the adult, adolescent, and child populations.

## 2 Materials and methods

### 2.1 Sample collection

**Fig. 1:**
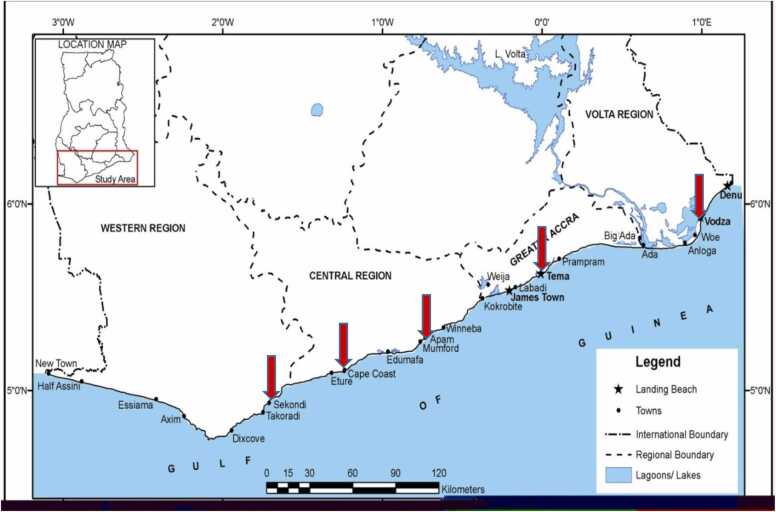
Map of the Coastline of Ghana showing the five fish landing beaches sampled.

Freshly landed *S. maderensis* (Madeiran sardinella)*, S. sphyraena* (Mediterranean barracuda)*, D. angolensis* (Angolan Dentex) *and P. notialis* (Southern pink shrimp) from Ghanaian coastal waters were obtained from five (5) major fish landing beaches between June and September 2017 for the study (Fig. 1). As fish are highly migratory organisms, we consider fish landed in these major landing sites to represent fish caught from the nearshore and offshore areas of the Ghanaian EEZ as well as those of neighboring countries such as Cote D′Ivoire, Togo, Benin and Nigeria. These fish species are among the most frequently landed species along the sub-region with huge economic potential [36]. A total of 200 individual fishes (10 individuals per specie at each landing site) were used for the study. Fish specimens were washed with deionized water to remove adhering particles, wrapped in sterile polyethylene bags, and transported on ice to the Wet Laboratory of the Department of Marine and Fisheries Science, University of Ghana for preparation. Sample preparation was performed in accordance with USEPA Guide No. 823-B-00–007 [13], [14].

### 2.2 Sample preparation

In the laboratory, vital information such as weight and standard length were used in computing the condition factors of the different fish species (Table 1). Fish samples were placed on a PTFE cutting board and scales were removed by scrapping from the tail to the head using the blade edge of a titanium knife. Cross contamination was avoided by rinsing the blade with deionized water between fish. Exactly 40 g of muscle tissue was removed from each fish of the same species from each landing beach to form a composite sample (200 g per composite of 5). The samples were freeze-dried at - 80 °C for 48 h to constant weight and homogenized in a high-speed stainless-steel blender. Prepared samples were stored in sealed air-tight plastic bags and later digested and analyzed at the Research Centre for Toxic Compounds in the Environment, Masaryk University, Brno, Czech Republic.

**Table 1:** Morphometrics and Ecology of the fish species analyzed.

Species	Standard length (cm) Mean ± SD Range		Weight (g) Mean ± SD Range		Condition Factor (k)	Habitat	Feeding habit
*Sardinella maderensis*	16.2 ± 0.4	15.8–17.0	73.8 ± 3.3	68.5–79.4	1.74	Pelagic	Herbivorous
*Dentex angolensis*	17.2 ± 0.8	15.9–18.0	129.4 ± 6.2	110.1–159.4	1.16	Demersal	Omnivorous
*Penaeus notialis*	11.8 ± 0.7	10.6–13.2	38.4.24 ± 3.7	35.5–42.2	2.33	Benthic	Herbivorous
*Sphyraena sphyraena*	21.5 ± 1.1	20.1–23.1	115.2 ± 4.5	102–128	2.54	Semi pelagic	Carnivorous

### 2.3 Sample digestion and analyses

The content of Cu, Zn, As, Cd and Pb in each fish tissue was determined after microwave digestion (MWS 3 + Berghof system, Germany) of the samples. Approximately 300 mg of each composite sample was digested with concentrated nitric acid (4 ml, Merck p.a.) and hydrogen peroxide (2 ml, Merck p.a.) followed by inductively coupled plasma mass spectrometry (Agilent 7700x ICP-MS, Japan). The Hg content of each composite sample was analyzed directly without any treatment using an AMA254 mercury analyzer (Altec s.r.o, Czech Republic). Method validation was carried out by analyzing certified reference materials (DORM-2 Dogfish Muscle, NRC-CNRC), spiked samples and performing method blanks (Table 2). Typical relative standard deviations for replicate analysis are reported in single units of percent.

**Table 2:** Quality assurance of trace metal analysis determined by certified reference material DORM-2 Dogfish Muscle, NRC-CNRC.LOQs and spiking recoveries.

Elements	Limits of Quantification (mg/kg)	Measured Concentration (mg/kg)	Certified Concentration (mg/kg)	Spiking recoveries (%)
Pb	0.3	< 0.3	0.065 ± 0.007	101 ± 1
Cu	0.08	2.07 ± 0.08	2.34 ± 0.16	96 ± 6
Zn	2.	26.4 ± 2.3	25.6 ± 2.3	97 ± 2
As	0.004	17.3 ± 0.8	18.0 ± 1.1	108 ± 6
Cd	0.0006	0.043 ± 0.003	0.043 ± 0.008	99 ± 1
Hg	0.0003	4.38 ± 0.08	4.64 ± 0.26	a/n

### 2.4 Statistical analysis

The data obtained were statistically analyzed using the statistical package, IBM SPSS 24.0 (SPSS, USA). The means and standard deviations of metal concentrations in fish species were recorded. One Way Analysis of Variance (ANOVA) and Multivariate post hoc Tukey tests was used in examining the statistical significance of the differences in mean concentrations of trace metals among different fish species. The level of significance was set at p < 0.05.

### 2.5 Human health risk assessment

#### 2.5.1 Estimated daily intake of metal (EDI)

The dietary exposures to trace metals for children, adolescents, and adults were estimated using the average trace metal concentrations determined for each fish species. For EDI and other health risk parameter estimations, the established average body weights for the three different age categories were 21 kg for children aged 6 years, 51 kg for adolescents aged 14 years, and 70 kg for adults aged 70 years [55]. The estimated daily intake of each metal per meal of fish consumed was estimated for the various age categories using Eq. 1 [8]. The estimated daily intake (EDI) values obtained were compared to the provisional permissible tolerable daily intake (PTDI) values established by the Joint FAO/WHO Expert Committee on Food Additives [22].

(1) $$\mathrm{EDI}=\frac{\mathrm{FIR}\times C}{\mathrm{BW}}$$

Where, FIR is the fish ingestion rate, set at 76.7 g/ person/ day for Ghana [21]: *C* is the metal concentration in fish [ug/g w.w)] and BW is the average body weight of the different age groups ranging from 21 kg to 70 kg.

#### 2.5.2 Non-carcinogenic risk

The non-carcinogenic health risk was estimated using the Target Hazard Quotient equation developed by the USEPA. The Target Hazard Quotient (THQ) is defined as the ratio of the lifetime average daily dose to the reference dose (RfD) [12]. The target hazard quotient (THQ) does not predict the actual adverse health effect on the exposed population but offers a signal of the risk level due to pollutant exposure. If the THQ value is below 1, the exposed population may not experience any adverse health effects. However, if the THQ value is greater than one (THQ > RfD), the exposed population will most likely experience adverse health effects. The higher the THQ value, the higher the probability of the hazard to the exposed population. The THQ was estimated from Eq. 2 [16].

(2) $$\mathrm{THQ}=\frac{\mathrm{EF}\times \mathrm{ED}\times \mathrm{FIR}\times C}{\mathrm{RfD}\times \mathrm{BW}\times \mathrm{TA}}$$

Where EF is exposure frequency (365 days/year); ED is exposure duration for the three different age categories. The ED was set at 6 years for children, 14 years for adolescents and 70 years for the adult population (. FIR is the fresh fish ingestion rate for Ghana (76.7 g/ person/day); C is the metal concentration in fish (µg/g ww); RfD is the oral reference dose which represents the daily intake of a contaminant that is not expected to cause adverse health effects over a lifetime. THQ was estimated using oral reference doses of 3.0 × 10^−1^ mg kg^−1^d^−1^ for Hg, 4.0 × 10^−2^ mg kg^−1^d^−1^ for Cu, 3.0 × 10^−1^ mg kg^−1^d^−1^ for Zn, 3.0 × 10^−4^ mg kg^−1^d^−1^ for As and 1.0 × 10^−3^ mg kg^−1^d^−1^ for Cu [16]. BW is the average body weight (21 kg for children, 51 kg for adolescents and 70 kg for adults) and TA is the average exposure time for non-carcinogens (365 days/year × ED). In the Target hazard quotient estimation, it is assumed that the ingestion dose is equal to the adsorbed contaminant dose and that cooking has no effect on the contaminants [28]. For the cumulative risk of trace metal contamination in fish, a total hazard index (HI) was used. The hazard index (HI) from THQs is expressed as the sum of the hazard quotients.

(3) HI=THQ (Hg) +THQ (Cd) +THQ (Cu) +THQ (As) +THQ (Zn)

#### 2.5.3 Carcinogenic risk (CR)

For carcinogens, risks were estimated as the incremental probability of individuals developing cancer over a lifetime due to exposure to a potential carcinogen [17]. The CR was computed using Eq. 4 [15].

(4) $$\mathrm{CR}=\frac{\mathrm{EF}\times \mathrm{ED}\times \mathrm{FIR}\times C\times \mathrm{CSFo}}{\mathrm{BW}\times \mathrm{TA}}$$

Where: CSFo is the cancer slope factor (ug/g/day) from the Integrated Risk Information System database, which is 1.5 (mg/kg/day) ^−1^ for inorganic arsenic [13], [14].

The THQ and CR risk calculations for As were made on the assumption that the toxic inorganic arsenic was 3% of total As [53], [8].

## 3 Results and discussion

### 3.1 Trace metals in fish species

The mean concentrations and standard deviations of trace metals in S. maderensis, D. angolensis, S. sphyraena, and P. notialis from Ghanaian coastal waters are presented in Table 3 below. With the exception of Pb, which was below the detection limit (0.3 μg/g) in all fish species analyzed, Cu, Zn, Hg, Cd, and As were detected in all fish species. The crustacean P. notialis recorded the highest concentrations of Cu, Zn, As, and Cd, whereas S. sphyraena recorded relatively low concentrations of these metals. Significant differences were observed (p = 0.001) in metal concentrations across all four fish species. These variations are expected because the accumulation of heavy metals in fish depends on several factors, including the concentration of the metal in the water, time of exposure, the uptake process, environmental conditions (temperature, pH, dissolved oxygen), and intrinsic factors such as age, feeding habits, and habitat [29]. The crustacean P. notialis is brackish and benthic, whereas D. angolensis is a marine and demersal fish. Both S. sphyraena and S. maderensis are pelagic-neritic marine species. Generally, heavy metal levels were about twofold higher in benthic and demersal fish compared to pelagic species. This trend is similar to that observed in the South China Sea [45], where it was observed that sediments contribute more to the accumulation of heavy metals in fish than in water. Epibenthic fishes and crustaceans that spend most of their lives on the sediment floor feed primarily in the sediment, absorbing more heavy metals that bind to fine sediment particles.

**Table 3:** Mean metal concentration (µg/g ww) ± SD of trace metals in assessed species.

Fish species	Cu (mg/kg)	Zn (mg/kg)	Cd (mg/kg)	As (mg/kg)	Hg (mg/kg)
*S. maderensis*	1.17 ± 0.14	10.41 ± 1.16	0.005 ± 0.002	1.56 ± 0.24	0.021 ± 0.004
*D. angolensis*	0.31 ± 0.06	5.54 ± 0.95	0.006 ± 0.003	1.87 ± 0.52	0.137 ± 0.021
*P. notialis*	12.08 ± 1.02	19.21 ± 1.54	0.027 ± 0.014	8.48 ± 2.04	0.026 ± 0.005
*S. sphyraena*	0.33 ± 0.04	4.51 ± 0.27	0.002 ± 0.001	0.82 ± 0.31	0.063 ± 0.011
Permissible Limit	30^a^	30 – 50^a^	0.05^b^	2.0^c^	0.5^b^

a [19]

b [10]

c FSANZ 2008

Copper levels in the fish species ranged from 0.31 μg/g to 12.08 μg/g. The levels are below the FAO [19] permissible seafood intake level of 30 μg/g and are comparable to those recorded in pelagic fish species from the Mediterranean and Black Sea Regions [40], [43], [54]. Zinc concentrations in the fish species ranged from 4.23 μg/g to 19.21 µg/g and followed the order *S. sphyreana* < D. *angolensis* < *S. maderensis* < *P. notialis.* These levels were within the FAO recommended permissible seafood intake level of 30 µg/g, implying no immediate adverse health effect. Arsenic concentrations in the fish species ranged from 0.8 μg/g to 8.5 μg/g and followed the order: *S.sphyreana* < *S.maderensis* < *D. angolensis* < *P. notialis.* Arsenic levels in P. notialis largely exceeded the permissible seafood intake levels of 2.0 μg/g and 1.3 µg/g set by both the Food Standard Australia New Zealand- [24] and the USEPA, respectively (USEPA 2000), implying both short-term and long-term adverse health effects (Table 3). The exceedingly high levels of As in the fish species suggest a potential As pollution problem in Ghanaian and African coastal waters, as previous studies reported elevated levels in the ranges of 5.7 μg/g to 94.0 μg/g and 45 μg/g to 142 μg/g in marine and estuarine environments of Ghana, respectively [38], [39], as well as high levels (0.02 and 1760 μg L^−1^) in groundwater with values ranging up to 10,000 μg L^−1^ in surface water from different parts of Africa [3]. Arsenic pollution in Ghanaian waters has been attributed to the mobilization of As through arsenopyrite oxidation in groundwater as well as through mining activities and the use of arsenic-based herbicides in agricultural processes [51]. The concentration of Cd in the fish species ranged from 0.002 μg/g to 0.027 μg/g and followed the order S. sphyraena < S. maderensis < D. angolensis < P. notialis. The levels of Cd in all four fish species were within the permissible seafood intake level of 0.05 μg/g set by the European Commission [10]. Cadmium concentrations obtained in this study for all four fish species were also lower than those obtained in other pelagic and demersal fish species in the Mediterranean and the Black Sea Regions [40], [54], Sarkar et al. [48] from Bangladesh, Kaya and Turkoglu [31] from Turkey and Olmedo et al. [44] from Spain. Contrary to the other metals, Hg, ranged in concentration from 0.021 μg/g to 0.137 μg/g and followed the order S. maderensis < P. notialis < S. sphyreana < D. angolensis, as opposed to P. notialis, which recorded the highest levels of all the other metals. Mercury concentration was, however, within the permissible limit of 0.5 μg/g recommended by the European Commission [10]. Penaeus notialis and D. angolensis are benthic and demersal fishes, respectively, hence, we attribute the relatively higher concentrations of heavy metals recorded in these two species to the fact they live in or near sediments, which act as natural sinks for heavy metals and other contaminants in the marine environment [46], [5]. As size and age play a crucial role in the bioaccumulation and magnification of Hg, we attributed the high levels of Hg in D. angolensis to its size (Table 1).

### 3.2 Estimation of potential human health risks

Although the levels of all trace metals, except for As, did not exceed permissible seafood intake limits, their actual toxicity depends on exposure doses, frequencies, and durations. Exposure doses, frequencies, and durations vary from individual to individual and according to age. Therefore, estimating daily consumption is considered a tool for evaluating the balance between benefits and risks. The EDIs calculated in this study were compared to the provisional maximum tolerable daily intake (PTDI) (g/person/day) established by the FAO/WHO Expert Committee on Food and Additives. Except for As, which exceeded the set limit in all age groups (Table 4), all metals are within the PTDI, implying that exposure to these metals has no potential health effects.

**Table 4:** Estimated Daily Intake (EDI) in Adults (a), Adolescents (b) and Children (c) compared with provisional permissible tolerable daily intake.

PTDI: provisional permissible tolerable daily intake (μg/kg body weight/day), FAO/WHO [22]

**Fig. 2:**
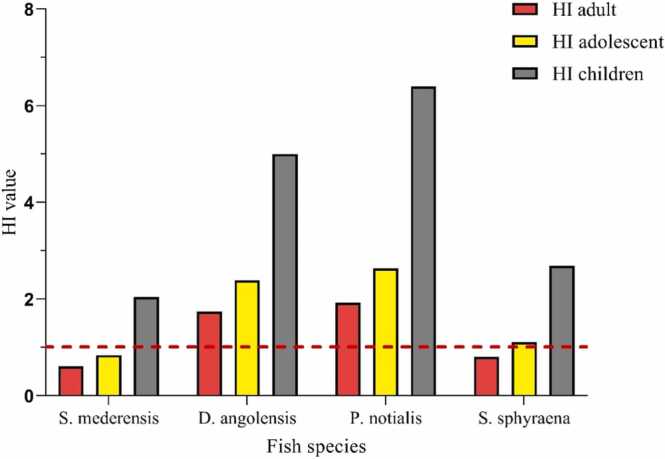
Health Risk Index (HI) calculation for heavy metals in fish species.

While computed EDIs suggest no risk to all age groups for Hg and Cu exposures, the THQ of Hg exceeded 1 for D. angolensis in all 3 age groups (Table 5), while that of As for P. notialis exceeded 1 in both adolescents and children. Likewise, the THQ of Cd for P. notialis exceeded 1 in children. These observations imply that children and adolescents are the most likely to experience significant non-carcinogenic health risks from the intake of Hg, Cd, and As in these fish species in the long term. The results of this study, therefore, support the European Commission’s restrictions concerning the consumption of marine species (especially predatory species) by children, pregnant women, and breastfeeding mothers [10]. The Hazard Index (HI), which describes the combined effect of multiple trace metal consumption from a fish, was greater than 1 in all age populations for *P. notialis* and *D. angolensis* (Fig. 2). This implies that the consumption of these fish species may result in some non-carcinogenic effects.

**Table 5:** Estimated Target Hazard Quotient (THQ) in Adults (a), Adolescents (b) and Children (c) from heavy metal exposure.

THQ > 1 is highlighted in red

The target carcinogenic risks (CRs) derived from the intake of As were computed because the consumption of As may promote both non-carcinogenic and carcinogenic effects depending on the exposure dose. The CRs were computed using the concentration of inorganic As because this form of the metal is a classified known carcinogen (USEPA Group A). Although Hg (methylmercury) and Cd are categorized in USEPA groups C (possible carcinogens to humans due to animal evidence and little or no human data) and B1 (probable carcinogen due to human studies), respectively, their CRs were not computed because Ingestion Slope Factors (CSFo) are not established yet for these elements [16]. For carcinogens, the USEPA sets a target risk level of 10^−6^ to denote negligible cancer risks for individual chemicals [16]. It is, however, recommended that the cumulative cancer risks for all potential carcinogenic contaminants do not have a residual cancer risk exceeding 10^−4^ [16]. From this study, cancer risks due to the intake of As from all fish species exceeded the 10^−6^ threshold for children age group (Table 6) as well as exceeding the threshold for *S. maderensis* across the adolescent group.

**Table 6:** Cancer risk estimate for inorganic As, assumed as the 3% of the total concentrations, for different age groups.

CR > 10^−5^ is highlighted in red

Despite the health implications associated with the consumption of the assessed fish from the coast of Ghana, we acknowledge that the sample size is relatively small, and so it is recommended that further studies be undertaken to confirm these findings.

## 4 Conclusions

Heavy metal pollution is a global problem that has implications for ecosystem functioning, global food safety and security. While extensive studies on trace metal levels in seafood have been carried out in many parts of the world to guide seafood consumption, little or no studies exist from other parts of the world including the Gulf of Guinea Region. The study, therefore, comes to fill an important gap in knowledge by providing information on heavy metal levels in four (4) commonly consumed fish species from the Gulf of Guinea and their possible implications to human health.

Except for As, the levels of all the metals studied were below the permissible limits set by various regulatory authorities in the world. Arsenic levels were above permissible limits in all fish species except *S. sphyreana*. Even though, the concentrations of Cu and Hg, and their computed EDIs pose no apparent health threats, the THQs due to exposures to these metals and As implied possible manifestations of significant non-cancer related health risks in children and adolescents. Non-cancer risks due to As and Cu intake are higher for the consumption *P. notialis* while those due to Hg intake are higher for *D. angolensis* and *S. sphreana* consumption*.* The study further reports significant carcinogenic risks due to As intake from *S. maderensis* and *D. angolensis* in most and in all age categories for *P. notialis*.

We therefore strongly recommend that to avoid the manifestations of non-carcinogenic risks, the consumption of these fish species by adolescents, children, pregnant women, and nursing mothers is done with caution while measures are taken to reduce their levels in the marine environment. While further studies are recommended to corroborate these conclusions, caution should be taken in the consumption of *P. notialis* to avoid likely carcinogenic risks in children, adolescent, and adult populations.

## CRediT authorship contribution statement

**Elvis Nyarko**: Conceptualization, Writing – review & editing, Supervision, Funding acquisition. **Charles Mario Boateng**: Conceptualization, Methodology, Investigation, Writing – original draft, Visualization. **Maurice Oti Edusei**: Conceptualization, Methodology, Writing. **Obed Asamoah**: Conceptualization, Writing – review & editing. **Edem Mahu**: Conceptualization, Methodology, Writing – review & editing, Supervision, Formal analysis.

## Declaration of Competing Interest

The authors declare that they have no known competing financial interests or personal relationships that could have appeared to influence the work reported in this paper.

## Data Availability

Data will be made available on request.
